# Tumor Burden of Iodine-Avid Bone Metastatic Thyroid Cancer Identified via ^18^F-Sodium Fluoride PET/CT Imaging

**DOI:** 10.3390/jcm13020569

**Published:** 2024-01-19

**Authors:** Carmela Nappi, Emilia Zampella, Valeria Gaudieri, Fabio Volpe, Leandra Piscopo, Carlo Vallone, Leonardo Pace, Andrea Ponsiglione, Simone Maurea, Emanuele Nicolai, Alberto Cuocolo, Michele Klain

**Affiliations:** 1 Department of Advanced Biomedical Sciences, University of Naples Federico II, Via Pansini 5, 80131 Napoli, Italyvaleria.gaudieri@gmail.com (V.G.); leandra.piscopo@unina.it (L.P.); carlovallone15@gmail.com (C.V.); cuocolo@unina.it (A.C.); micheleklain@libero.it (M.K.); 2 Department of Medicine, Surgery and Dentistry, University of Salerno, 84084 Salerno, Italy; lpace@unisa.it; 3 IRCCS Synlab-SDN, 80143 Naples, Italy; emanuele.nicolai@synlab.it

**Keywords:** PET/CT, SPECT, WBS, ^18^F-NaF, ^18^F-FDG, ^131^I, DTC, bone metastases

## Abstract

Background: Patients with differentiated thyroid cancer (DTC) are referred to radioactive ^131^I (RAI) therapy and post-therapy ^131^I whole-body scintigraphy (WBS) to identify local and/or remote metastases. Positron emission tomography (PET)/computed tomography (CT) imaging with ^18^F-fluoro-D-glucose (FDG) or ^18^F-sodium fluoride (NaF) may also be used with these patients for the evaluation of bone metastases. We compared the role of ^18^F-NaF PET/CT and ^18^F-FDG-PET/CT in patients with DTC and documented bone metastases at post-therapy WBS. Methods: Ten consecutive DTC patients with iodine avid bone metastasis at post-therapy WBS referred to ^18^F-NaF PET/CT and ^18^F-FDG PET/CT were studied. The findings of the three imaging procedures were compared for abnormal detection rates and concordance. Results: At post-therapy ^131^I WBS, all patients had skeletal involvement with a total of 21 bone iodine avid lesions. At ^18^F-FDG PET/TC, 19 bone lesions demonstrated increased tracer uptake and CT pathological alterations, while 2 lesions did not show any pathological finding. At ^18^F-NaF PET/CT, the 19 bone lesions detected at ^18^F-FDG PET/TC also demonstrated abnormal tracer uptake, and the other 2 bone iodine avid foci did not show any pathological finding. Conclusions: In patients with DTC, ^18^F-NaF PET/CT did not obtain more information on the metastatic skeletal involvement than post-therapy ^131^I WBS and ^18^F-FDG PET/CT.

## 1. Introduction

Differentiated thyroid cancer (DTC) is the most frequent endocrine malignancy, and its rates are raising all around the world [1]. It is believed that this increase is mainly due to better screening procedures, such as the introduction of neck ultrasounds in daily practices as part of the basic assessment of thyroid diseases. According to histological staging, after surgery, patients with DTC can be led to radioactive (RAI) therapy with ^131^I for ablation purposes in protected hospitalization. After 5–9 days since RAI, a ^131^I whole-body scintigraphy (WBS) is performed to identify local and/or distant metastases greedy for iodine. Follow-ups consist of periodical instrumental and biochemical analyses of patients in suppressive therapy with thyroid hormones [1]. As part of the monitoring of DTC patients undergoing RAI, the surveillance program includes the evaluation of thyroglobulin (Tg) levels, anti-Tg antibodies and neck ultrasounds for the early detection of loco-regional recurrence [2]. Once the Tg blood level rises and remote metastases are suspected, a positron emission tomography (PET)/computerized tomography (CT) with 2-deoxy-2-[18F]-fluoro-D-glucose (^18^F-FDG) is performed to identify any metastatic foci. In addition to cervical lymph nodes, the most frequent localizations of repetitive DTC lesions are the lung and skeleton. Although PET/CT with ^18^F-FDG has high sensitivity in identifying lesions with high metabolic turnover, an earlier marker of bone involvement would allow more effective and less invasive therapeutic approaches before bone damage inexorably affects the quality of life [3]. The positron-emitting radiopharmaceutical ^18^F-NaF was introduced decades ago for skeletal imaging [4]. Currently, ^18^F-NaF PET/CT is used for the functional imaging of pathologies with high osteogenic metabolism. This diagnostic procedure is used for the detection and localization of bone metastases in cancer patients [5,6,7,8]. Prior studies suggest that ^18^F-NaF PET/CT imaging may provide high sensitivity and specificity in the detection of bone metastases [9,10,11]. A study carried out in 52 oncological patients demonstrated the superiority of PET/CT with ^18^F-NaF over bone scintigraphy with ^99m^Tc methyl-diphosphonate (MDP) in terms of image quality and a more accurate evaluation of the extent of skeletal disease [12]. Schirrmeister et al. [13] confirmed the better performance of ^18^F-NaF PET/CT over ^99m^Tc-MDP bone scintigraphy in DTC patients with skeletal metastases. Yet, only a few data are available on the comparison between ^18^F-FDG PET/CT and ^18^F-NaF PET/CT in oncological patients [14,15]. The aim of the present pilot investigation was to assess the role of ^18^F-NaF PET/CT in evaluating patients with DTC and known bone metastases at post-therapy ^131^I WBS undergoing ^18^F-FDG-PET/CT.

## 2. Methods

### 2.1. Study Popolation

Ten consecutive patients were recruited prospectively from June 2020 to November 2022. The inclusion criteria were as follows: (a) age older than 18 years; (b) DTC previously treated with total thyroidectomy and post-operative ^131^I administration; (c) presence of iodine-avid bone metastases at post-therapy WBS with indication for ^18^F-NaF PET/CT to evaluate further skeletal involvement; and (d) suspicion of non-iodine avid metastases with indication to conduct a ^18^F-FDG PET/CT scan according to serum Tg and Tg antibody levels. Exclusion criteria were (a) pregnancy, (b) blood glucose levels greater than 140 mg/dL (7.77 mmol/L), (c) inability to tolerate the scan due to claustrophobia or pain and (d) previous external beam treatment on bone metastatic lesions. The protocol was approved by the Ethics Committee of the National Cancer Institute of Naples (Protocol numbers 2–11) and was performed in accordance with the guidelines of the Helsinki II declaration. All participants signed an informed consent form before being included in the study.

### 2.2. Imaging

Post-therapy WBS was conducted 7 days after the administration of a therapeutic dose of ^131^I (1850–5550 MBq). All patients received serum thyroid-stimulating hormone (TSH) concentrations of 30 µIU/mL or more at the time of ^131^I administration. Planar images were obtained using a dual-head γ-camera (E.CAM, Siemens Medical Systems, Hoffman Estates, IL, USA) equipped with a high-energy collimator. Data analysis was performed by two nuclear medicine physicians on a dedicated workstation. The WBS results were considered positive when at least one abnormal focus of ^131^I uptake was found. All pathological foci were recorded and noted. For the purpose of the study, only patients with iodine-avid bone metastases were referred to further imaging investigations. ^18^F-FDG PET/CT whole-body scans were performed 45 to 60 min after tracer administration (3.7 MBq/kg). All patients fasted for at least 6 h prior to imaging, and blood glucose levels were <180 mg/dL at the time of tracer injection. Images were obtained using a PET/CT scanner (Gemini TF 64 scanner, Philips Healthcare, Best, The Netherlands). A CT scan for attenuation correction (average parameters 80 kV, 40 mA) was acquired before PET for a total imaging time of 20 min. A ^18^F-NaF PET/CT whole-body scan was obtained 90 min after tracer administration (2.6 ± 1.0 MBq/kg). Images were obtained using a PET/CT system (Discovery IQ, GE Healthcare Discovery IQ, GE Healthcare, Chicago, IL, USA). A diagnostic CT scan for fusion was obtained using a standard protocol without intravenous contrast (120 kV; Auto mA range, 30–250 mA, thickness 3.75 mm). All scans were performed in 3-dimensional mode. PET data were reconstructed with and without attenuation correction into transverse, sagittal and coronal images with a standard iterative algorithm using software provided by the equipment manufacturers that considered attenuation, detector efficiency, scatter and random coincidence corrections. PET images were classified as follows: areas of focally increased tracer uptake corresponding to pathological CT findings including those showing mixed or lytic patterns, with or without sclerotic margins or sclerotic CT features with benign patterns or absent CT alterations.

### 2.3. Statistical Analysis

Data were expressed as mean ± standard deviation or as percentages. Weighted kappa values (Cohen’s coefficient) were calculated to measure the degree of agreement between imaging methods.

## 3. Results

### 3.1. Clinical Characteristics

The clinical characteristics of the study population are shown in Table 1. Nine patients were on LT4 withdrawal at the time of RAI, while one patient underwent the rh-TSH protocol before RAI therapy due to comorbidity. Tg levels at time of RAI therapy ranged from 77 to 13,098 ng/dL with a mean of 2441 ± 4048 ng/dL (mean thyroid-stimulating hormone: 66 ± 36 mUI/mL). Tg antibody levels were unremarkable in all patients. Only two patients received the first RAI dose at the time of investigation, and they had bone metastases diagnosed by a pathologist’s report. The other eight patients had previously received at least one RAI therapy (range 1–5), and ^131^I was formerly administered once to four of them. All of these eight patients had biochemically and structurally persistent metastatic disease, with evidence of RAI-avid metastases prior post-therapy WBS. All patients were treated for metastatic disease therapy purposes.

### 3.2. Imaging Findings

Post-therapy WBS revealed skeletal involvement with a total of 21 iodine-avid bone foci across all patients (Table 2). In particular, five patients presented a singular bone lesion; three patients, two bone lesions; and two patients, ≥3 bone metastatic foci. Regarding extra-skeletal observations, only one patient had residual thyroid bed uptake, while two patients had thyroid bed recurrence; three patients, latero-cervical or mediastinal iodine-avid nodes; and five patients, lung uptake (Table 3).

**Figure 1:**
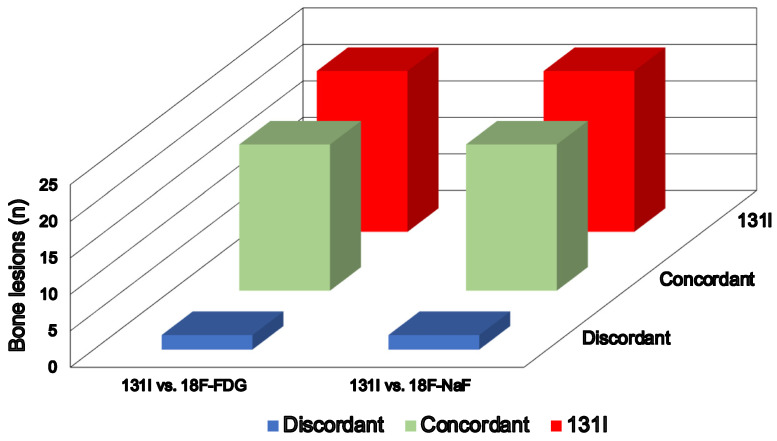
Comparison of bone lesions detected via post-therapy ^131^I WBS with ^18^F-FDG and ^18^F-NaF PET/CT.

At ^18^F-FDG PET/TC, 19 bone lesions in nine patients demonstrated focal tracer uptake and CT abnormalities. Conversely, the two foci with increased ^131^I activity at post-therapy WBS in the remaining patient did not show any ^18^F-FDG focal pathological uptake. Regarding the extra-skeletal findings, the residual thyroid tissue, demonstrating ^131^I uptake at post-therapy WBS, did not show any ^18^F-FDG accumulation, while the two thyroid bed recurrences and iodine-avid nodes exhibited significant metabolic activity. Of the five patients with lung involvement, ^18^F-FDG uptake was observed in only three of them. However, it is essential to consider that the lung nodules in the other two patients were less than 5 mm in size at CT, rendering them metabolically not assessable. At ^18^F-NaF PET/CT, the same 19 bone foci identified with ^18^F-FDG PET/TC exhibited abnormal tracer uptake. Similarly, the remaining two bone avid foci did not show any pathological tracer accumulation (Figure 1), and there were no notable CT findings, achieving a 100% agreement (K coefficient, 1) between the two PET/CT methods in assessing bone metastasis. Additionally, another six focal ^18^F-NaF bone uptake sites were observed in five patients. However, these findings showed benign patterns at CT, attributed to arthrosis degeneration. Of note, both PET scans were not able to identify bone metastatic lesions in addition to those already identified at post-therapy WBS (K coefficient, 0.95).

**Figure 2:**
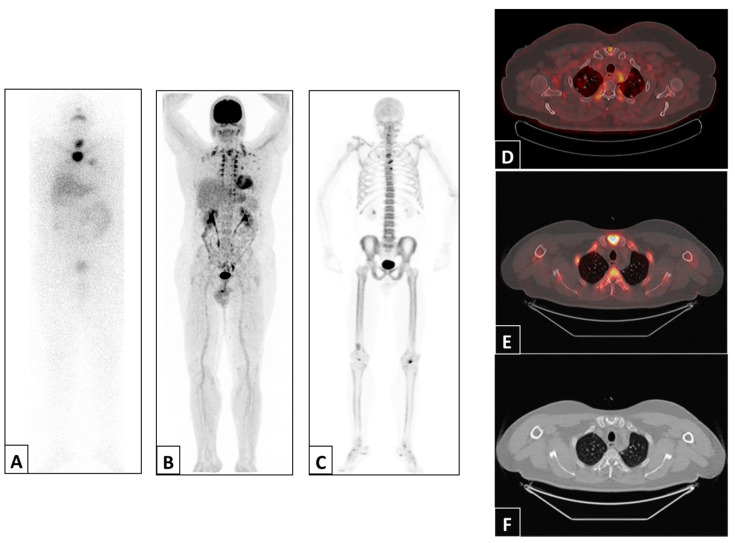
Representative example of a patient with abnormal uptake in the sternum detected via post-therapy ^131^I WBS anterior projection (**A**), ^18^F-FDG PET/CT maximum intensity projection (**B**) and ^18^F-NaF PET/CT maximum intensity projection (**C**) and ^18^F-FDG PET/CT (**D**), ^18^F-NaF PET/CT (**E**) and CT (**F**) trans-axial views of the sternal manubrium.

**Figure 3:**
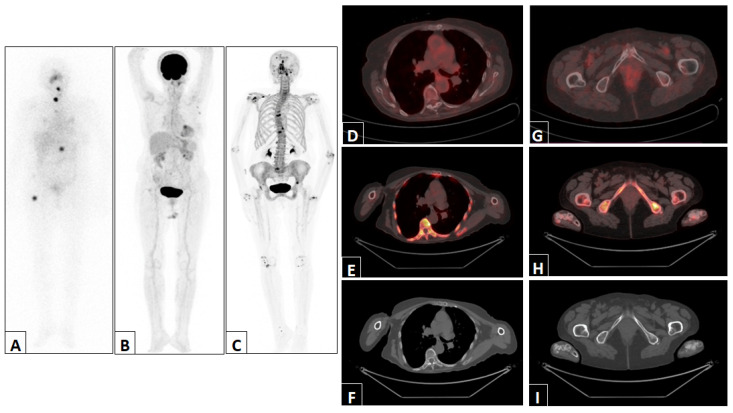
Representative example of a patient with abnormal uptake in D12 and the right femur detected via post-therapy ^131^I WBS anterior projection (**A**). The ^18^F-FDG PET/CT maximum intensity projection (**B**), ^18^F-NaF PET/CT maximum intensity projection (**C**) and ^18^F-FDG PET/CT, ^18^F-NaF PET/CT and CT trans-axial views of D12 (**D**–**F**) and the right femur (**G**–**I**) were normal.

Representative examples of imaging findings in two patients are depicted in Figure 2 and Figure 3.

## 4. Discussion

In our patient population, 21 bone iodine-avid lesions were detected via post-therapy ^131^I WBS. The large majority (19) of these lesions also demonstrated ^18^F-FDG- and ^18^F-NaF-increased uptakes along with CT alterations. The remaining two bone iodine-avid lesions detected in the same patient were not noticed either at the metabolic or morphological level by either scan. However, these findings should be read in the light of the elevated Tg levels in a patient undergoing their first RAI treatment, confirming the strong diagnostic value of high-dose ^131^I imaging in DTC in the early identification of metastatic involvement. Additionally, another six focal ^18^F-NaF bone uptake sites were observed in five patients with an arthrosis degeneration pattern. The relatively young age of the majority of patients (6 out of 10) is consistent with the observation of only six degenerative lesions with focal ^18^F-NaF uptake. Therefore, our results suggest that the use of ^18^F-NaF did not obtain more information on the skeletal metastatic burden than post-therapy ^131^I WBS and ^18^F-NaF PET/CT. From a dosimetry point of view, it should be considered that ^18^F-NaF PET/CT implies that a similar amount of ^18^F-FDG PET/CT is an effective dose with a cost-effectiveness-increased price. Indeed, while ^18^F-NaF imaging may provide information regarding only the bone metastatic load, the use of a ^18^F-FDG scan may deliver a more complete evaluation of disease staging [16], including nodes, lung and other extra-skeletal compartments also offering a true complement to post-therapy ^131^I WBS for the evaluation of tumor burden involvement [17,18,19]. Moreover, when integrated with magnetic resonance imaging, ^18^F-FDG PET may offer a more accurate evaluation of soft tissue compartments with lower radiation exposure [20]. According to prior studies [21,22], the areas of increased ^18^F-NaF uptake that were not observed on the ^131^I scan demonstrated sclerotic morphological characteristics, confirming that the method features high sensitivity but low specificity. Increased ^18^F-NaF uptake in bone may have different etiologies [21,22,23]. Indeed, the level of ^18^F-NaF activity is not strictly correlated with the malignant nature of the finding, and benign lesions may demonstrate an even prominent ^18^F-NaF accumulation.

A co-registered CT scan may lead to the correct interpretation of the integrated imaging data. CT morphological evaluations are still a benchmark with a sensitivity of 73% and specificity of 95 for oncological applications [24]. Nevertheless, structural alterations became evident only after pathological metabolic changes [25]. Thus, the combined utilization of high-dose ^131^I imaging as an early marker of disease advancement and the use of a CT scan to guide morphological assessment may be considered the best method for DTC theragnostic management, and also for the evaluation of bone involvement [26].

To the best of our knowledge, the present investigation is the first to explore the role of ^18^F-NaF PET/CT on bone evaluations in patients with DTC undergoing RAI therapy and ^18^F-FDG PET/CT with known skeletal involvement as assessed via post-therapy ^131^I WBS. The application of the ^18^F-NaF PET/CT tool on oncological grounds has been considered in patients with different tumors [6,22,27,28,29]. In comparison with bone scintigraphy, ^18^F-NaF imaging had boasted the potential to replace ^99m^Tc-MDP as a reference bone imaging tool in a phase 3 trial conducted on 290 patients with high-risk prostate or breast cancers [30]. Yet, Piccardo and co-workers [21] found that while ^18^F-FDG PET/CT has independent prognostic implications in patients with breast cancer and bone metastases, ^18^F-NaF PET/CT does not achieve independent predictive values even with a higher diagnostic accuracy than that of ^18^F-FDG PET/CT. Conversely, Ueda et al. [26] observed that in 31 patients with medullary thyroid cancer, ^18^F-NaF demonstrated equivalent or higher diagnostic power compared to other imaging modalities in the detection of whole-body skeletal metastases.

It must be pointed out that the current survey considers only patients with DTC and iodine-avid bone metastases. Thus, the role of ^18^F-NaF PET/CT in iodine-refractory DTC patients has not been explored yet, and the possibility for ^18^F-NaF PET/CT to identify bone metastatic DTC patients before post-therapy ^131^I WBS should be further investigated. This issue still represents an unmet need. On the other hand, between the RAI-avid and non-avid patients’ categories, there is a population with both ^131^I and ^18^F-FDG uptakes in the same lesion or in different lesions representing an assorted group of patients with tumor heterogeneity, and similar prognosis compared to the only ^18^F-FDG uptake group [31,32]. However, from the present investigation, it can be reasonable that ^18^F-NaF PET/CT may not add any information for the identification of skeletal involvement over WBS and ^18^F-FDG, even in the heterogeneous disease group. A potential opportunity in this context could be provided by ^68^Ga-DOTA-FAPI-04. A recent study demonstrated that this radiopharmaceutical is a promising molecule for diagnosing, opening up the way to radioligand therapy in iodine-refractory DTC patients [33].

Of note, it should be highlighted that patients with previous external beam treatment on bone metastatic lesions were not enrolled in this study. Radiation therapy determines bone hypoxia, hypocellularity and the reduction of vascularization bringing down the osteoblastic and osteoclastic metabolisms [34]. All these effects, taken together, may lead to a decrease in radiopharmaceuticals bone uptake including ^18^F-NaF. Other limitations should also be considered. Our investigation refers to a small population size. Moreover, the sample was also heterogeneous in terms of treatment, as different doses were applied as well as different numbers of doses received, and all this can influence the number of lesions that can be seen and vice versa as there was no control group to compare with. On the other hand, the presented preliminary data are not encouraging to go on recruitment with additional radiation dose administration to perform further ^18^F-NaF PET-CT in this category of patients without the potential for a significant improvement in clinical management. In addition, follow-up data are not available yet. Thus, the prognostic value of the ^18^F-NaF method still needs to be assessed. Finally, only planar ^131^I imaging was available at the time of the study, and hybrid ^131^I scintigraphy integrated with CT data would have improved the accuracy of the current work.

## 5. Conclusions

The results of this study demonstrate that ^18^F-NaF PET/CT does not obtain additional information on metastatic skeletal involvement in DTC patients undergoing post-therapy ^131^I WBS and ^18^F-FDG PET/CT. Furthermore, post-therapy WBS and PET/CT with ^18^F-FDG allowed the evaluation of other potential disease compartments such as the thyroid bed, lymph nodes and lungs, confirming the higher disease detection rates of these approaches compared to other imaging modalities. However, the potential use of ^18^F-NaF PET/CT in patients with iodine-refractory DTC suggests that further investigations are needed to better understand its role in DTC patients with bone metastatic disease.

## Figures and Tables

**Table 1:** Clinical characteristics of the study population.

Pt	Age (Years)	Sex	Histology	Stage	Tg (ng/dL)	TSH (30 µIU/mL)	Activity (MBq)	RAI (*n*)
1	55	F	FV-PTC	pT3NxM1	90	77	3700	4
2	51	M	PTC	pT3bNxM1	147	33	5550	2
3	51	F	FV-PTC	pT3bNxM1	77	52	3700	3
4	58	F	FTC	pT2NxM1	461	32	3700	2
5	65	M	PTC	pT1(m)Nx	2245	36	3700	5
6	65	F	FTC	M1	4306	113	7400	1
7	86	F	FTC	pT3NxMx	199	30	3700	2
8	71	F	FV-PTC	Pt2(m)N0M1	3383	120	5550	4
9	78	M	FV-PTC	pT2NxM1	13,098	75	6785	2
10	81	F	FTC	pT3N0Mx	403	100	3700	1

PTC, Papillary thyroid carcinoma; FTC, Follicular thyroid carcinoma; FV-PTC, Follicular variant of papillary thyroid carcinoma; Tg, Thyroglobulin; TSH, Thyroid-stimulating hormone; RAI, radioactive iodine.

**Table 2:** Skeletal imaging findings in the study population.

Pt	Lesion Site	Post-Therapy WBS	^18^F-FDG PET/CT	^18^F-NaF PET/CT	CT
1	L5	+	+	+	Lytic with sclerotic margins
	Right femur	+	+	+	Lytic with sclerotic margins
2	Sternum	+	+	+	Mixed
3	Right femur	+	+	+	Mixed
4	L3	+	+	+	Lytic
5	Frontal bone	+	+	+	Lytic
6	Sternum	+	+	+	Lytic with sclerotic margins
	L1	+	+	+	Lytic
	L5-S1	+	+	+	Lytic
7	Left ischiopubic branch	+	+	+	Lytic
8	Left iliac wing	+	+	+	Lytic
	Right sacral wing	+	+	+	Lytic with sclerotic margins
9	Right sphenoid	+	+	+	Lytic
	Sternum	+	+	+	Lytic
	III left rib	+	+	+	Lytic
	Left humerus	+	+	+	Lytic
	Right iliac plug	+	+	+	Lytic
	Right pubic bone	+	+	+	Lytic
	Right acetabular cavity	+	+	+	Lytic
10	D12	+	−	−	−
	Right femur	+	−	−	−

**Table 3:** Extra-skeletal imaging findings in the study population.

Pt	Lesion Site	Post-Therapy WBS	^18^F-FDG PET/CT
1	Lungs	+	+
	Thyroid bed recurrence	+	+
2	Lungs	+	+
	Thyroid bed recurrence	+	+
	Mediastinal lymph nodes	+	+
3	Mediastinal lymph nodes	+	+
4	−	−	−
5	Right lung	+	−
6	−	−	−
7	Right lung	+	+
8	Left lung	+	−
9	Mediastinal lymph nodes	+	+
10	Thyroid residue	+	−

## Data Availability

The data presented in this study are available on request from the corresponding author. The data are not publicly available due to privacy restrictions.
